# Required sample size to detect mediation in 3-level implementation studies

**DOI:** 10.1186/s13012-022-01235-2

**Published:** 2022-10-01

**Authors:** Nathaniel J. Williams, Kristopher J. Preacher, Paul D. Allison, David S. Mandell, Steven C. Marcus

**Affiliations:** 1 Institute for the Study of Behavioral Health and Addiction, Boise State University, 1910 University Drive, Boise, ID 83725-1940 USA; 2 School of Social Work, Boise State University, Boise, ID USA; 3 Department of Psychology & Human Development, Vanderbilt University, 230 Appleton Place, Nashville, TN 37203-5721 USA; 4 Statistical Horizons LLC, P.O. Box 282, Ardmore, PA 19003 USA; 5 Penn Center for Mental Health, University of Pennsylvania School of Medicine, 3535 Market Street, Philadelphia, PA 19104 USA; 6 Department of Psychiatry, University of Pennsylvania School of Medicine, 3535 Market Street, Philadelphia, PA USA; 7 School of Social Policy & Practice, University of Pennsylvania, 3701 Locust Walk, Philadelphia, PA 19104-6214 USA

**Keywords:** Statistical power, Indirect effects, Mediation, Multilevel, Mplus

## Abstract

**Background:**

Statistical tests of mediation are important for advancing implementation science; however, little research has examined the sample sizes needed to detect mediation in 3-level designs (e.g., organization, provider, patient) that are common in implementation research. Using a generalizable Monte Carlo simulation method, this paper examines the sample sizes required to detect mediation in 3-level designs under a range of conditions plausible for implementation studies.

**Method:**

Statistical power was estimated for 17,496 3-level mediation designs in which the independent variable (*X*) resided at the highest cluster level (e.g., organization), the mediator (*M*) resided at the intermediate nested level (e.g., provider), and the outcome (*Y*) resided at the lowest nested level (e.g., patient). Designs varied by sample size per level, intraclass correlation coefficients of *M* and *Y*, effect sizes of the two paths constituting the indirect (mediation) effect (i.e., *X*→*M* and *M*→*Y*), and size of the direct effect. Power estimates were generated for all designs using two statistical models—conventional linear multilevel modeling of manifest variables (MVM) and multilevel structural equation modeling (MSEM)—for both 1- and 2-sided hypothesis tests.

**Results:**

For 2-sided tests, statistical power to detect mediation was sufficient (≥0.8) in only 463 designs (2.6%) estimated using MVM and 228 designs (1.3%) estimated using MSEM; the minimum number of highest-level units needed to achieve adequate power was 40; the minimum total sample size was 900 observations. For 1-sided tests, 808 designs (4.6%) estimated using MVM and 369 designs (2.1%) estimated using MSEM had adequate power; the minimum number of highest-level units was 20; the minimum total sample was 600. At least one large effect size for either the *X*→*M* or *M*→*Y* path was necessary to achieve adequate power across all conditions.

**Conclusions:**

While our analysis has important limitations, results suggest many of the 3-level mediation designs that can realistically be conducted in implementation research lack statistical power to detect mediation of highest-level independent variables unless effect sizes are large and 40 or more highest-level units are enrolled. We suggest strategies to increase statistical power for multilevel mediation designs and innovations to improve the feasibility of mediation tests in implementation research.

**Supplementary Information:**

The online version contains supplementary material available at 10.1186/s13012-022-01235-2.

Contributions to the literature

- Multilevel mediation analysis is an important tool for testing mechanisms in implementation science; however, little is known about the sample sizes required to adequately power these studies particularly within the range of sample sizes that are feasible for implementation research
- We calculated statistical power to detect mediation in 3-level designs (e.g., organization, provider, patient) using a range of plausible input values and sample sizes for implementation research
- Less than 5% of designs had adequate statistical power to detect mediation; large effect sizes and samples of 40 or more clusters (e.g., organizations) were typically required
- Results indicate changes are needed in how mechanisms are studied in implementation science and in the expectations of research funders

## Background

**Fig. 1:**
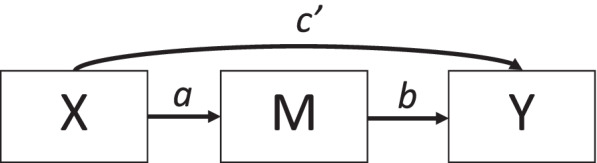
Single-level mediation model. *Note: X* = independent variable; *M* = mediator, *Y* = outcome. The indirect effect is estimated as the product of the *a* and *b* paths (i.e., a*b). The *c’* path represents the direct effect of *X* on *Y* (i.e., the effect of *X* on *Y* that is not transmitted through the mediator, *M*)

The goal of implementation science is to improve the quality and effectiveness of health services by developing strategies that promote the adoption, implementation, and sustainment of empirically supported interventions in routine care [1]. Understanding the causal processes that influence healthcare professionals’ and participants’ behavior greatly facilitates this aim [2, 3]; however, knowledge regarding these processes is in its infancy [4, 5]. One popular approach to understanding causal processes is to conduct mediation studies in which the relationship between an independent variable (*X*) and a dependent variable (*Y*) is decomposed into two relationships—an indirect effect that occurs through an intervening or mediator variable (*M*) and a direct effect that does not occur through an intervening variable [6, 7]. Figure 1 shows a mediation model in which the effect of *X* on *Y* is decomposed into direct (*c’*) and indirect effects (the product of the *a* and *b* paths). Estimates of the *a*, *b*, and *c’* paths shown in Fig. 1 can be obtained from regression analyses or structural equation modeling. Under certain assumptions, these estimates allow for inference regarding the extent to which the effect of *X* on *Y* is mediated, or transmitted, through the intervening variable *M* [8–10]. Interpreted appropriately, mediation analysis enables investigators to test hypotheses about *how X* contributes to change in *Y* and thereby to elucidate the mechanisms of change that influence implementation [5, 9, 10]. Recently, several major research funders, including the National Institutes of Health in the USA, have emphasized the importance of an experimental therapeutics approach to translational and implementation research in which mechanisms of action are clearly specified and tested [11–13]. Mediation analysis offers an important method for such tests.

Mediation analysis has long been of importance in implementation science, with recent studies emphasizing the need to increase the frequency and rigor with which this method is used [5, 14]. Guided by theoretical work on implementation mechanisms [15, 16], emerging methods-focused guidance for implementation research calls for the use of mediation analyses in randomized implementation trials to better understand how implementation strategies influence healthcare processes and outcomes [5, 17]. A systematic review of studies examining implementation mechanisms indicated mediation analysis was the dominant method for testing mechanisms in the field, used by 30 of 46 studies [4]. Other systematic reviews highlight deficits in the quality of published mediation analyses in implementation science to date and have called for increased and improved use of the method [5, 18]. Reflecting its growing importance within the field, mediation analyses feature prominently in several implementation research protocols published in the field’s leading journal, *Implementation Science*, during the last year [19–22]. Chashin et al. [23] recently published guidance for reporting mediation analyses in implementation studies, including the importance of determining required sample sizes for mediation tests a priori.

Designing mediation studies requires estimates of the sample size needed to detect the indirect effect. This seemingly simple issue takes on special nuance and heightened importance in implementation research because of the complexity of statistical power analysis for multilevel research designs—which are the norm in implementation research [17, 24]—and the constraints on sample size posed by the practical realities of conducting implementation research in healthcare systems. While statistical power analysis methods and tools for single-level mediation are well-developed and widely available [8, 25–29], these approaches are inappropriate for testing mediation in studies with two or more hierarchical levels, such as patients nested within providers nested within organizations [9, 30, 31]. Generating correct inferences about mediation from multilevel research designs requires multilevel analytic approaches and associated power analyses to determine the required sample size [32–36].

While some tools have begun to emerge to estimate required sample sizes for 2- and 3-level mediation designs [37, 38], findings from this preliminary research indicate that calculation of statistical power for multilevel mediation is complex and depends on the anticipated range and configuration of study design input values—such as effect sizes and sample sizes—at each level (e.g., organization, clinician, patient). As a result, the feasibility of obtaining adequate sample sizes to test multilevel mediation is highly field-dependent; which mediation hypotheses can be realistically tested in implementation science depends on the anticipated range and configuration of realistic study design input values for the field. In implementation research, resource and practical constraints often limit the sample sizes that are feasible to recruit and enroll at the highest level of the design—for example, the number of geographical areas, organizations, or clinics that can be studied—thus potentially restricting the mediation hypotheses that can be realistically tested. Furthermore, the structure of healthcare systems and natural constraints on healthcare processes (e.g., patient flow) often limit the number of providers available within higher-level units over a project period as well as the number of patients each provider serves. These field-specific constraints on sample sizes at each level create a more specific and high-stakes question for implementation scientists interested in using mediation analysis: what are the minimum sample sizes required—at each level—to detect mediation in 3-level designs, *given what is realistic for implementation settings*?

### Mediation analysis in multilevel studies

Krull and MacKinnon describe multilevel mediation designs by the level of each variable in the *X*→*M*→*Y* chain [33]. Each level in the design represents a different level of sampling (e.g., organization, clinician, provider) and units at lower levels (e.g., patients) are assumed to be nested within units at higher levels (e.g., clinicians). For example, organizations may be at the highest level (level 3), clinicians may be nested within organizations (level 2), and patients may be nested within clinicians (level 1).

**Fig. 2:**
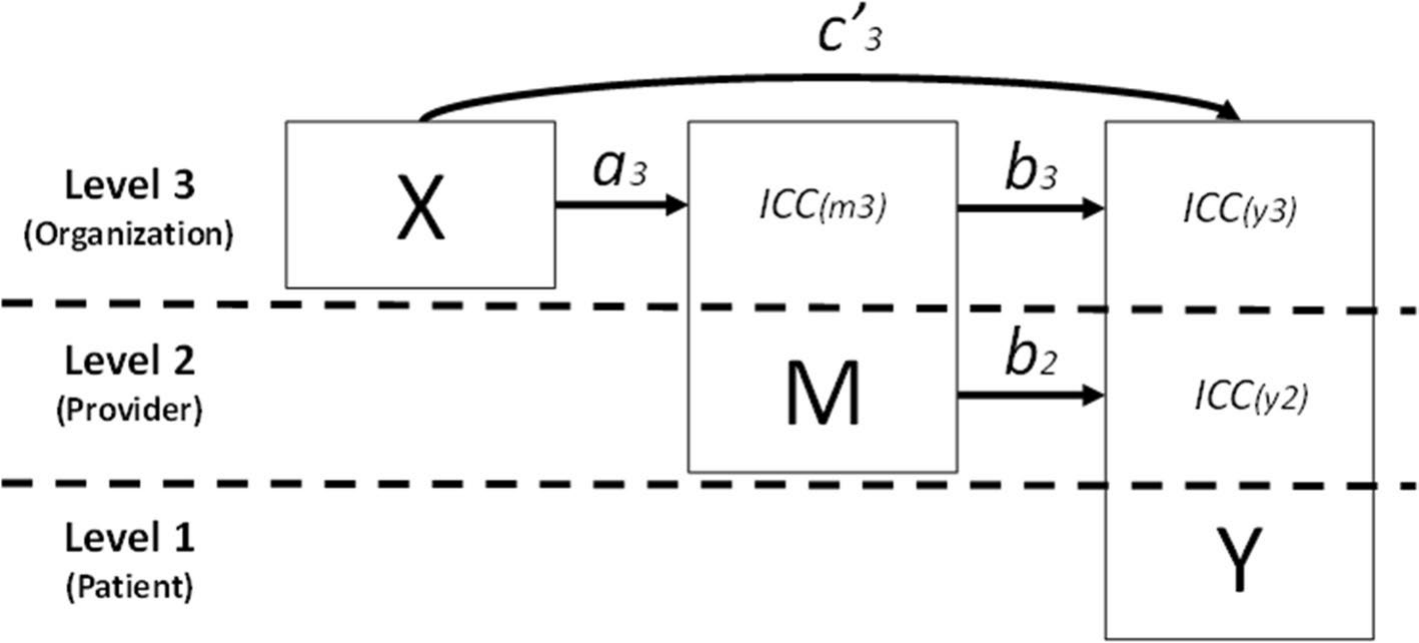
Multilevel mediation model (3-2-1). *Note:* The diagram presents the 3-2-1 mediation design for which statistical power was calculated in this study. The boxes signify each construct in the design and show the levels at which the construct exhibits variance: *X* = independent variable which varies only at level 3; *M* = mediator which resides at level 2 but exhibits variance at levels 2 and 3 (due to clustering); *Y* = outcome which resides at level 1 but exhibits variance at levels 1, 2, and 3 (due to clustering). The variance of *M* and *Y* at the higher levels of analysis are represented by ICC values. Arrows indicate effects that can be estimated through conventional multilevel regression (MVM) [32] or through multilevel structure equation modeling (MSEM) [36]. The paths that make up the indirect effect (i.e., mediation at level 3) are *a*_3_*b_*3*_. The *c’*_*3*_ path represents the direct effect. The *b*_*2*_ path is typically not of substantive interest; it represents the relationship between the within-organization component of *M* and within-organization component of *Y*

Figure 2 presents a conceptual model of a 3-level mediation design and the parameter values the investigator must supply to estimate statistical power or the required sample size. Similar to a protocol by Aarons et al. [39], in this example, an organization-level implementation strategy (*X*) at level 3, is designed to influence a patient-level implementation outcome at level 1 (*Y*) through its effects on a level 2 clinician mediator (*M*). The *X* variable is random assignment to an organizational implementation strategy versus a control condition. Aarons et al. [39] describe a strategy that trains organizational leaders in skills and strategies that improve clinicians’ implementation citizenship behaviors. Increases in clinicians’ implementation citizenship behavior (level 2 *M*) is hypothesized to increase patients’ experience of high-fidelity care (level 1 *Y*). In the figure, these relationships correspond to the *a*_*3*_ and *b*_*3*_ paths, respectively, which make up the indirect effect at level 3. The *c’*_*3*_ path represents the direct effect.

To estimate statistical power for this example, the investigator must supply (1) alpha level (typically set at *α*=0.05); (2) 1- vs. 2-sided hypothesis test; (3) sample size for each level; (4) standardized effect sizes for the *a*_*3*_, *b*_*3*_, and *c’*_*3*_ paths at level 3; (5) a standardized effect size for the *b*_*2*_ path at level 2; and (6) values of the intraclass (or intracluster) correlation coefficient (ICC) for the mediator *M* at level 3 (*ICC*_*m3*_) and, for the outcome *Y*, at levels 2 (*ICC*_*y2*_) and 3 (*ICC*_*y3*_). The ICC is a ratio describing the proportion of variance in a variable that resides at each level of the design [40]; it can be interpreted as the extent to which observations within a cluster are correlated with one another [30]. In this example, *ICC*_*y3*_ represents the variance of the outcome *Y* that occurs between organizations (e.g., the variance in the means of *Y* across organizations), and *ICC*_*y2*_ represents the variance of the outcome that occurs between clinicians *within* organizations [40]. *ICC*_*m3*_ represents the variance of the mediator *M* that occurs between organizations.

In multilevel designs, one can test mediation hypotheses using two different statistical approaches: traditional multilevel modeling based on manifest (i.e., observed) variables (MVM) or multilevel structural equation modeling (MSEM). MVM approaches test mediation based on observed data using traditional multilevel models [32], which are sometimes referred to as hierarchical linear models [30] or mixed effects models [31]. Many software programs provide routines to analyze data using these models [34]. MSEM uses structural equation modeling to partition observed variables into latent components at different levels of the design and subsequently tests mediation using these latent components [35, 36]. Analogous to the relationship between linear regression and single-level structural equation modeling [41], MSEM represents a large-sample approach to multilevel mediation analysis that engenders greater modeling flexibility and produces more accurate effect estimates relative to MVM at the cost of higher standard errors and lower statistical power [35, 42–44].

### Study contributions and aims

**Fig. 3:**
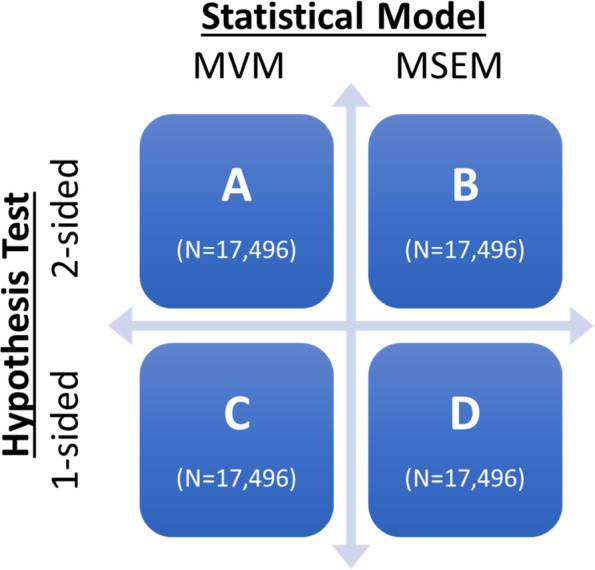
Statistical models and types of hypothesis tests studied. *Note:* We conducted statistical power simulations for 17,496 implementation research designs under four different conditions. The conditions represent a fully crossed matrix of two different statistical models (traditional multilevel modeling of manifest variables [MVM] and multilevel structural equation modeling [MSEM]) and two different hypothesis tests for the mediation effect (1- and 2-sided).

In this study, we address the issue of statistical power and minimum sample sizes required to test mediation in 3-level implementation studies using a generalizable method for calculating statistical power based on Monte Carlo simulations. We examined statistical power for mediation in 17,496 3-level designs that varied across a range of design parameter input values deemed plausible for implementation research in healthcare settings. As is shown in Fig. 3, power was estimated for all designs using two statistical models: MVM (cells A and C) and MSEM (cells B and D) for both 2-sided (cells A and B) and 1-sided (cells C and D) hypothesis tests.

Our study makes four contributions to implementation science. First, our power analyses address a specific range of realistic design parameter input values for implementation studies in healthcare. As such, our results represent a useful resource and potentially cautionary note for implementation scientists planning multilevel mediation studies. Second, our simulation-based approach to determining statistical power overcomes the limitations of prior formula-based work [38] that does not address power for MSEM designs. While some tools are available to estimate statistical power for multilevel mediation in 2- [37] and 3-level trials [38], these approaches do not accommodate MSEM designs. Often, they accommodate cluster randomized trials but not observational studies. By providing our simulation code to investigators, we offer a power analysis template for multilevel mediation that addresses MSEM for 3-level observational or cluster randomized designs that can be easily modified for 2-level designs. Third, our approach overcomes the limitations of formula-based tools for 3-level mediation designs which make the restrictive and unrealistic assumption that the direct effect is zero (e.g., 38). This is important because direct effects are rarely equal to zero in implementation studies (see 5) and because non-zero direct effects meaningfully influence statistical power and sample size in 3-level designs (as is shown below). Fourth, our simulation-based approach incorporates sufficient flexibility to allow investigators to revise the code to address hypotheses regarding moderated mediation (i.e., effect modifiers) and other design variations which are not possible with the limited formula-based tools currently available for 2- or 3-level mediation designs [45].

Focusing on design parameters that are realistic for implementation studies in healthcare, the research questions were as follows: (1) How many of the plausible designs studied had adequate statistical power to detect mediation? (2) What study characteristics were associated with increased statistical power to detect mediation? (3) What was the range of minimum required sample sizes to detect mediation within this set of plausible designs? We provide our code in Additional file 1 as a resource for investigators to estimate statistical power for designs not examined here.

## Method

Our method for estimating statistical power was based on empirical Monte Carlo simulations [46, 47]. Under this approach, many samples of a specified size are generated from a hypothetical population and the model of interest is estimated in each sample. Statistical power is computed as the proportion of samples (e.g., 400 out of 500) in which the parameter of interest is statistically significant. Monte Carlo simulation methods are well-established as a general approach to determining statistical power; they make similar assumptions as formula-based approaches but have greater flexibility for estimating power in complex models derived from hierarchically selected samples [46, 47]. We followed guidelines for reporting Monte Carlo simulation studies as suggested by Boomsma [48].

We used simulations to estimate statistical power for designs that incorporated a continuous outcome and mediator and varied systematically with regard to the population design parameters shown in Fig. 2. Values for each of the nine design parameters were fully crossed, resulting in 17,496 designs (3^7^*4*2). Following prior work [29, 49], values of the two standardized paths that make up the indirect effect (i.e., *a*_*3*_ and *b*_*3*_) were set at 0.14, 0.39, and 0.59, which represent small (~2% of the variance), medium (~13% of the variance), and large (~26% of the variance) effect sizes, respectively^1^, as suggested by Cohen [50]. Based on the same logic, values of the standardized *c’*_*3*_ path, which represents the direct effect, were set at 0.14 (small) and 0.39 (medium). Values of the standardized *b*_*2*_ path, which is not typically of substantive interest in implementation studies, were fixed at 0.39 (medium). Values of ICC for the mediator and outcome were set at 0.05, 0.10, and 0.20 at each relevant level of the design. These correspond to small, medium, and large ICCs based on research describing ranges of ICC for process and endpoint variables in implementation research and healthcare settings [51–53].

We studied a range of sample sizes relevant to implementation research. As is shown in Table 1, the level-3 sample size (*N*_*3*_) represents the number of highest-level clusters (e.g., organizations), the level-2 sample size (*N*_*2*_) represents the number of intermediate-level units per cluster (e.g., providers), and the level-1 sample size (*N*_*1*_) represents the number of lowest-level units per intermediate unit (e.g., patients). Guided by the range of sample sizes observed in systematic reviews of implementation studies [5, 54–57], level-3 sample sizes were set at 10, 20, 40, and 60. We chose 10 because it was the expected lower limit on the number of level-3 units (e.g., organizations) necessary to achieve adequate power and 60 because reviews of implementation studies suggest 60 is often the largest feasible sample size. Level-2 sample sizes were set at 5, 10, and 20, reflecting a minimum number of intermediate-level units (e.g., providers) expected to achieve adequate power and an upper limit expected to reflect larger samples in healthcare settings. Level-1 sample sizes were set at 3, 6, and 12, reflecting a minimum number of lowest-level units (e.g., patients) to justify clustering and an anticipated upper limit feasible to recruit during a time-limited period. The code in Additional file 1 can be modified to calculate power for designs not studied here.

**Table 1:** Frequency of study designs with statistical power ≥ 0.8 by study characteristic (*N* = 17,496 designs)

Study characteristic	Parameter value	Total ***N*** of designs	***N*** of adequately powered designs (≥ .8)	Proportion of adequately powered designs (% ≥.8)
Total		17,496	463	2.6%
*a*_*3*_ (standardized *X*→*M* coefficient)	0.14	5832	0	0.0%
	0.39	5832	46	0.8%
	0.59	5832	417	7.2%
*b*_*3*_ (standardized *M*→*Y* coefficient)	0.14	5832	0	0.0%
	0.39	5832	32	0.5%
	0.59	5832	431	7.4%
*c’*_*3*_ (standardized direct effect)	0.14	8748	161	1.8%
	0.39	8748	302	3.5%
*ICC*_*M3*_ (level-3 intraclass correlation coefficient for mediator *M*)	0.05	5832	36	0.6%
	0.10	5832	125	2.1%
	0.20	5832	302	5.2%
*ICC*_*Y2*_ (level-2 intraclass correlation coefficient for outcome *Y*)	0.05	5832	136	2.3%
	0.10	5832	148	2.5%
	0.20	5832	179	3.1%
*ICC*_*Y3*_ (level-3 intraclass correlation coefficient for outcome *Y*)	0.05	5832	140	2.4%
	0.10	5832	161	2.8%
	0.20	5832	162	2.8%
*N*_*3*_ (level-3 sample size; *N* of highest-level units/clusters, e.g., organizations)	10	4374	0	0.0%
	20	4374	0	0.0%
	40	4374	110	2.5%
	60	4374	353	8.1%
*N*_*2*_ (level-2 sample size; *N* of nested intermediate-level units per cluster, e.g., providers)	5	5832	42	0.7%
	10	5832	129	2.2%
	20	5832	292	5.0%
*N*_*1*_ (level-1 sample size; *N* of nested lowest-level units per intermediate unit, e.g., patients)	3	5832	131	2.2%
	6	5832	159	2.7%
	12	5832	173	3.0%
Total sample size (*N*_3_* N_2_* N_*1*_)	150	486	0	0.0%
	300	1458	0	0.0%
	600	2916	0	0.0%
	900	486	9	1.9%
	1200	3402	8	0.2%
	1800	972	43	4.4%
	2400	2916	33	1.1%
	3600	1458	112	7.7%
	4800	1458	39	2.7%
	7200	972	116	11.9%
	9600	486	30	6.2%
	14,400	486	73	15.0%

*Note*: Power was calculated for *N* = 17,496 designs based on Monte Carlo simulations (500 replications per design) conducted in Mplus 8. All models represent 3-2-1 mediation designs estimated using maximum likelihood with robust standard errors based on a linear multilevel model with manifest variables (MVM). For each design, power was calculated as the proportion of replications (out of 500) for which the null hypothesis, *H*_*0*_: *a*_3_*b_*3*_ = 0, was rejected based on the Sobel test, assuming *α* = 0.05 (two-tailed)

For each design, 500 simulated datasets were generated using the MONTECARLO command in Mplus 8 [58]. These were analyzed using the TYPE=THREELEVEL option of the ANALYSIS command with the default maximum likelihood estimator with robust standard errors (MLR). Simulations were conducted on multi-processor computing platforms which allowed for simultaneous estimation of models.

We generated statistical power estimates for each of the 17,496 designs under four different conditions shown in Fig. 3. Cells A and C in Fig. 3 represent statistical power estimates generated for traditional multilevel models with manifest variables (MVM). Cells B and D represent statistical power estimates generated for MSEM. Indirect effects for MVM models were calculated using the “centered within context with means reintroduced” approach described by Zhang et al. [32]. MSEM indirect effects were based on latent partitioning of variables [35].

Cells A and B in Fig. 3 represent statistical power estimates for both MVM and MSEM using a 2-sided null hypothesis test (H_0_: *a*_3_*b_*3*_ = 0) with an alpha set at *α*=0.05. For these tests, we used the first-order delta method which is sometimes called the Sobel test [59]. The Sobel test is widely used for mediation analyses across multiple disciplines [6] and is slightly more conservative than computationally intensive bootstrapping methods [60] or the Monte Carlo confidence interval approach [26, 61].

Cells C and D in Fig. 3 represent statistical power estimates for MVM and MSEM using a 1-sided hypothesis test. Many mediation hypotheses could reasonably be specified as directional (i.e., 1-sided) because the implementation strategy is anticipated to have a positive (or negative) effect on the mediator and outcome. The use of a 1-sided test should reduce the sample size needed to detect mediation. Estimates of statistical power for 1-sided tests were generated using an algebraic transformation of the results from the 2-sided simulations and thus did not require additional computational time (details available upon request).

## Results

Completion of the simulations required 591 days of computational time. Completion rates, defined as the number of replications within a simulation that successfully converged (e.g., 500 out of 500), were high: 97.8% (*n*=17,114) of the MVM simulations exhibited complete convergence (i.e., 500 of 500 replications were successfully estimated) and 79.4% (*n*=13,889) of the MSEM simulations exhibited complete convergence. The lowest number of completed replications for any design was 493 (out of 500). The high rate at which the replications were completed increases confidence in the resulting simulation-based estimates of statistical power.

### How many of the designs studied had adequate statistical power to detect mediation?

Table 1 shows the frequency and percent of designs studied that had adequate statistical power (≥ 0.8) to detect mediation by study characteristic based on a conventional MVM model, using a 2-sided test (cell A in Fig. 3). Only 463 of the 17,496 (2.6%) designs had adequate statistical power to detect mediation. As expected, statistical power was higher for the designs in cell C of Fig. 3 which were estimated using MVM and a 1-sided hypothesis test: 808 of these designs (4.6%) had adequate power to detect mediation.

As an alternative to MVM, investigators may use MSEM. Focusing on cell B of Fig. 3 (MSEM, 2-sided test), results indicated that 228 of the 17,496 designs (1.3%) studied had adequate statistical power to detect mediation. Shifting to cell D of Fig. 3 (MSEM, 1-sided test): 369 of the designs (2.1%) had adequate statistical power.

In summary, less than 5% of the 3-level mediation designs studied had adequate statistical power to detect mediation regardless of the statistical model employed (i.e., MVM vs. MSEM) or whether tests were 1- vs. 2-sided.

### What study characteristics were associated with increased statistical power to detect mediation?

Table 1 presents the frequency and percent of designs with adequate statistical power to detect mediation by study characteristic for the 17,496 designs in cell A of Fig. 3 (MVM, 2-sided test). Because results were similar for all four cells in Fig. 3, we focus on the results from cell A and describe variations for the other cells as appropriate. Additional file 2 presents the frequency and percent of study designs with adequate statistical power to test mediation by study characteristic for all four cells shown in Fig. 3.

First, consistent with expectations, statistical power to detect mediation increased as the magnitude of effect sizes increased for the two paths that constitute the indirect effect (i.e., *a*_*3*_ and *b*_*3*_). Notably, none of the designs in Table 1 had adequate power when either the *a*_*3*_ or *b*_*3*_ paths were small; less than 1% of designs had adequate power when the *a*_*3*_ or *b*_*3*_ paths were medium.

Second, the number of adequately powered designs increased as sample sizes increased at each level, with the level-3 sample size having the largest effect on power. In Table 1, no designs with fewer than 40 level-3 clusters (e.g., organizations) had adequate power to detect mediation. This finding also held for the MSEM designs (cells B and D in Fig. 3; see Additional file 2). However, for cell C in Fig. 3 (MVM, 1-sided test), 11 designs (0.1%) had adequate power to detect mediation with level-3 sample sizes of 20 (see Additional file 2).

Third, larger total sample sizes were associated with increased power, although this relationship was not monotonic because the total sample size consisted of the product of the sample sizes at each level. In Table 1, the minimum total required sample size to detect mediation was *N*=900 level-1 units. The minimum total sample for cell C in Fig. 3 (MVM, 1-sided test) was *N*=600. The minimum total sample for cell B in Fig. 3 (MSEM, 2-sided test) was *N*=1800, and the minimum total sample for cell D in Fig. 3 (MSEM, 1-sided test) was *N*=1200.

### What was the range of minimum sample sizes required to detect mediation?

Table 2 presents the minimum sample sizes required to achieve statistical power ≥ 0.8 to detect mediation by values of effect size for the *a*_*3*_ and *b*_*3*_ paths that constitute the indirect effect, the size of the direct effect, and the level-3 ICCs of the mediator and outcome. Results in Table 2 are based on cell A of Fig. 3 (MVM, 2-sided). In each cell of Table 2, two sample sizes are provided, one assuming a small direct effect (*c*_*s*_) and the other assuming a medium direct effect (*c*_*m*_). Sample sizes are presented as *N*_*3*_ [*N*_*2*_ [*N*_*1*_]] where *N*_*3*_ = number of level-3 units (e.g., organizations), *N*_*2*_ = number of level-2 units (e.g., providers) per cluster, and *N*_*1*_ = number of level-1 units (e.g., patients) per level-2 unit. Because the *N*_*3*_ sample size is typically the most resource intensive to recruit in implementation studies, and because multiple combinations of *N*_*1*_, *N*_*2*_, and *N*_*3*_ can achieve the same total sample size in a given cell, the minimum sample sizes shown in Table 2 were selected based on the sample combination with adequate power and the smallest *N*_*3*_, followed by the smallest *N*_*2*_, followed by the smallest *N*_*1*_. Blank cells (-) are informative in that they indicate there were no sample sizes that achieved adequate statistical power to detect mediation for that design; for these cells, it is not possible to design a study with adequate statistical power to test mediation within the range of sample sizes and input values we tested. Additional file 3 provides a similar table for cell C of Figure 3 (MVM, 1-sided test).

**Table 2:** Minimum sample sizes required for adequate statistical power to detect mediation

*ICC*_*m3*_	*ICC*_*y3*_	Standardized effect sizes for *a*_*3*_ path (*X*→*M*) and *b*_*3*_ path (*M*→*Y*)								
		SS	SM	SL	MS	MM	ML	LS	LM	LL
S	S	-	-	-	-	-	-	-	-	*c*_*s*_: 60 [20 [6]]
										*c*_*m*_: 60 [20 [3]]
S	M	-	-	-	-	-	-	-	-	*c*_*s*_: 60[20[6]]
										*c*_*m*_: 60[20[3]]
S	L	-	-	-	-	-	-	-	-	*c*_*s*_: 60[20[6]]
										*c*_*m*_: 60[20[3]]
M	S	-	-	-	-	-	-	-	*c*_*s*_: -	*c*_*s*_: 40[20[12]]
									*c*_*m*_: 60[20[6]]	*c*_*m*_: 40[20[3]]
M	M	-	-	-	-	-	-	-	*c*_*s*_: -	*c*_*s*_: 40[20[6]]
									*c*_*m*_: 60[20[6]]	*c*_*m*_: 40[20[3]]
M	L	-	-	-	-	-	-	-	-	*c*_*s*_: 40[20[12]]
										*c*_*m*_: 40[20[3]]
L	S	-	-	-	-	-	*c*_*s*_: 60[20[3]]	-	*c*_*s*_: -	*c*_*s*_: 40[20[3]]
							*c*_*m*_: 60[20[3]]		*c*_*m*_: 60[10[12]]	*c*_*m*_: 40[10[3]]
L	M	-	-	-	-	-	*c*_*s*_: 60[20[3]]	-	*c*_*s*_: -	*c*_*s*_: 40[10[12]]
							*c*_*m*_: 60[10[12]]		*c*_*m*_: 60[10[6]]	*c*_*m*_: 40[10[3]]
L	L	-	-	-	-	-	*c*_*s*_: 60[20[3]]	-	*c*_*s*_: -	*c*_*s*_: 40[10[12]]
							*c*_*m*_: 60[10[12]]		*c*_*m*_: 60[10[6]]	*c*_*m*_: 40[5[6]]

*Note:* Sample sizes shown are the smallest sample size required to achieve statistical power ≥ 0.8 to reject the null hypothesis *a*_3_*b_*3*_ = 0 given the design parameters shown. Within each cell, two sample sizes are provided, one assuming a small direct effect (*c*_*s*_) and the other assuming a medium direct effect (*c*_*m*_). Sample sizes are presented as *N*_*3*_[*N*_*2*_[*N*_*1*_]] where *N*_*3*_ = number of highest-level clusters (level 3), *N*_*2*_ = number of intermediate nested units (level 2) per cluster, and *N*_*1*_ = number of lowest-level nested observations (level 1) per level-2 unit. Blank cells (-) indicate there were no sample sizes that achieved adequate power for that design. Required sample sizes were generated using linear multilevel modeling with manifest variables assuming *α*=0.05 (2-tailed). *ICC*_*m3*_ level-3 intraclass correlation coefficient of the mediator, *ICC*_*y3*_ level-3 intraclass correlation coefficient of the outcome. ICCs were evaluated at *S*=0.05, *M*=.1, and *L*=.2. Standardized effect sizes indicate the size of the *a*_*3*_ path followed by the size of the *b*_*3*_ path, where *S*=.14, *M*=.39, and *L*=.59

Table 2 provides additional insights into the design features necessary to test mediation in 3-level designs under conditions that are plausible for implementation research. First, most of the cells in Table 2 are empty, indicating no design in that cell had adequate power to detect mediation. This underscores the limited circumstances under which one can obtain a sample large enough to test mediation in 3-level implementation designs. Second, no designs with combinations of small or medium effects for the *a*_*3*_ and *b*_*3*_ paths had adequate statistical power. This indicates at least one large effect size for either the *a*_*3*_ or *b*_*3*_ path is needed to achieve adequate statistical power to test mediation. Third, the size of the level-3 ICC of the mediator (*ICC*_*m3*_) is extremely important. When *ICC*_*m3*_ is small, there are no designs with adequate power except those that have large effect sizes for both *a*_*3*_ and *b*_*3*_ paths.

## Discussion

Thought leaders and funders in the field of implementation science have increasingly called for a stronger focus on understanding implementation mechanisms [13–16], with methodologists pointing to mediation analysis as a recommended tool in this effort [5, 17]. Because statistical power to test mediation in multilevel designs depends on the specific range of input values that are feasible within a given research area, we estimated what sample sizes, effect sizes, and ICCs are required to detect mediation in 3-level implementation research designs. We estimated statistical power and sample size required to detect mediation using a range of input values feasible for implementation research. Designs were tested under four different conditions representing two statistical models (MVM vs. MSEM) and 1- versus 2-sided hypothesis tests (see Fig. 3). Fewer than 5% of the designs studied had adequate statistical power to detect mediation. In almost all cases, the smallest number of level-3 clusters necessary to achieve adequate power was 40, the upper limit of what is possible in many implementation studies. This raises important questions about the feasibility of mediation analyses in implementation research as it is currently practiced. Enrolling 40 organizations usually requires substantial resources and may not be feasible within a limited geographic area or timeframe [24, 55]. In many settings, it also may not be possible to enroll enough *level-2* units per setting (e.g., nurses on a ward, primary care physicians in a practice, specialty mental health clinicians in a clinic) or level-1 units (e.g., patients per provider). Below, we discuss the implications of these findings for researchers, funders of research, and the field.

### Implications for researchers

Implementation research commonly randomizes highest-level units to implementation strategies and measures characteristics of these units that may predict implementation, such as organizational climate or culture, organizational or team leadership, or prevailing policies or norms within geopolitical units. If researchers wish to study multilevel mediation, they must either obtain a large number of highest-level units or choose potential mediating variables that are likely to have large effects. While it is not known how often such level-3 independent variables have large effects on putative lower-level mediators, there are some encouraging data on the potential for large associations between lower-level mediators and lowest-level outcomes. For example, in a meta-analysis of 79 studies, Godin et al. found variables from social cognitive theories explained up to 81% of the variance in providers’ intention to execute healthcare behaviors and 28% of the variance in physicians’ behaviors, 24% of the variance in nurses’ behavior, and 55% of the variance in other healthcare professionals’ behavior [62]. These effect sizes are comparable to or larger than the effect size for the *b*_*3*_ path used in this study, suggesting that the variables proposed as antecedents to behavior in these theoretical models may serve as effective mediators linking level-3 independent variables to level-1 implementation outcomes.

Researchers can take steps to increase statistical power. One approach is to include a baseline covariate that is highly correlated with the outcome, ideally a pretest measure of the outcome itself, which can significantly increase statistical power, in some cases reducing the required sample size by 50% [30, 38, 63, 64]. The higher the correlation between the pretest covariate and the outcome, the lower the required sample size. Including a pretest of the mediator or outcome also increases the likelihood that the design meets the assumptions required to make causal inferences [65, 66]. However, whereas some settings like schools often have readily available pretests (e.g., academic achievement measures), pretests of implementation outcomes are not always available or may not make conceptual sense. For example, in implementation studies examining fidelity to a new practice, collecting pretest fidelity data may confuse participants because they have not yet learned the practice. Other approaches to increasing statistical power for indirect effects include using 1-sided hypothesis tests when appropriate [50], optimizing the reliability of measurement instruments [50], and using significance tests that are likely to engender higher statistical power, such as the distribution of the product method or Monte Carlo confidence intervals [26]. The chronic underuse of 1-sided hypothesis tests for indirect effects is puzzling considering they have significantly more power and are often justified by theory. Our results strongly support the use of 1-sided hypothesis tests for theory-informed multilevel mediation hypotheses.

### Implications for funders

Over the last decade, funding agencies like the US National Institutes of Health have made understanding the mechanisms by which interventions work part of funding announcements and the review process for implementation research [67]. The implications of this requirement, combined with other requirements that call for tests of mediation and moderation (i.e., sex as a biological variable; the role of treatment fidelity on outcome [68, 69]), place considerable demands on recruitment and measurement, even as the maximum budget for an R01 has not increased in almost 20 years. Funders may wish to change expectations for implementation research or emphasize trials that measure implementation outcomes but not clinical outcomes, which may allow for larger sample sizes at higher levels. Funders also may wish to develop funding mechanisms that provide additional funds to address the need for substantially larger sample sizes to test theories about mechanisms in multilevel contexts.

### Implications for the field

Our results are sobering and cause for reflection about how implementation science as a field approaches research designs that elucidate how our implementation strategies result in change. First, our results suggest the need for immediate studies to help researchers make sample size decisions. Because implementation science is a relatively new discipline, little data are available for estimating ICCs for outcomes at different levels. The field needs studies that summarize a wide range of ICCs for many implementation and clinical outcomes and for mediation targets across settings, populations, and interventions. The field also needs research that clarifies how different formal tests for mediation influence power in multilevel models. Although some studies have tested the performance of mediation tests in multilevel models [34, 38], much more work is needed. This line of research is especially important considering research from single-level models showing that some mediation tests display a better balance between type I error rates and statistical power [26].

Second, the field needs accurate measures of putative mediating variables. Increasing the accuracy of measurement will increase our ability to observe effects [50]. At present, the field does not have standardized ways to measure, for example, the constructs from cognitive theories often used as putative mediators [70]. The field could benefit from close collaboration with experts in those areas to develop agreed upon (and then tested) measurement strategies.

Third, the field should consider implementation strategies that are less expensive to implement. The expense of many implementation strategies has been documented in the literature, raising questions about scalability [71–73]. Less expensive strategies would increase our ability to test mechanism, but more importantly, increase the resources available to recruit more organizations into studies. Similarly, we should consider pragmatic trials that reduce measurement burden and allow us to enroll larger samples. Pragmatic trials differ from more traditional RCTs in that they can have more inclusive eligibility standards, the comparison condition, practitioner expertise and use of the intervention, primary outcome, and how these components are measured [74]. The focus of pragmatic trials is highly consistent with the goal of implementation science in understanding strategies to increase the use of evidence-based care in community practice and researchers have developed tools to describe the level of pragmatism in implementation trials [75].

### Study caveats and limitations

Our results indicate that investigators are unlikely to detect mediation in 3-level studies with samples of less than 40 highest-level units under conditions that are feasible in implementation science, although examples of positive studies may occur. In those cases, our results provide important context for interpreting the exceptional study’s results. First, low power to detect an effect does not mean it is impossible. Second, 3-level studies with samples of fewer than 40 highest-level units that do not detect mediation are likely never published, making the few published examples appear more common and representative than they are. Third, in some multilevel studies, indirect effects may be improperly specified and therefore statistically significant but not theoretically justified [32, 36, 76]. Fourth, studies may compensate for low *N*_*3*_ by having very large samples at other levels or higher effect sizes than those tested in our study.

The design parameters investigated in this study reflect a broad range of plausible values for 3-level designs in implementation research; however, there are undoubtedly important additional parameter values not studied here. We provide our code so investigators can study designs with other parameter values. The computational demands of bootstrapping and Monte Carlo confidence interval approaches led us to use the Sobel test for our study; consequently, power is likely to be slightly higher if investigators use these more powerful methods. Our study assessed mediation only in 3-2-1 designs that are broadly applicable to implementation science. Additional research should evaluate required sample sizes for power in other designs (e.g., 3-3-1, 3-1-1). To optimize potential generalizability and parsimony, our study did not include covariates in the mediation model; most notably, we did not include a pretest of the outcome. Covariates can reduce the required sample size to detect indirect effects [38] and future research is needed to characterize the types of pretest covariates that are available in implementation research as well as the strength of the relationship between these covariates and pertinent implementation and clinical outcomes as these will be important for study planning. Future research should also examine how unbalanced clusters influence power in multilevel mediation.

## Conclusions

This study assesses the sample sizes needed to test mediation in 3-level designs that are typical and plausible in implementation science in healthcare. Results suggest large effect sizes coupled with 40 or more highest-level units are needed to test mediation. Innovations in research design are likely needed to increase the feasibility of studying mediation within the multilevel contexts common to implementation science.

## Supplementary Information

**Additional file 1.** Mplus code for power analyses.

**Additional file 2.** Frequency of designs with adequate statistical power by method and test.

**Additional file 3.** Sample size crosstabulation.

## Data Availability

Additional file 1 provides Mplus code for running the simulations so investigators can recreate the results or produce new simulations for designs not studied here. Full results of the simulations are presented in Additional files 2 and 3.

## Abbreviations

EBP: Evidence-based practice
MSEM: Multilevel structural equation modeling
MVM: Conventional linear multilevel regression analysis using manifest (observed) variables

## Footnotes

1 Percentages in parentheses are approximate for the *b*_*3*_ and *c’*_*3*_ paths because they are partial coefficients.
