# Supplementary material for: Required sample size to detect mediation in 3-level implementation studies
Source: Implement Sci. 2022 Oct 1;17:66. doi: 10.1186/s13012-022-01235-2 (PMC9526963; doi:10.1186/s13012-022-01235-2)
Supplement: Supplementary file 1 — Additional file 1. Mplus code for power analyses. [file 13012_2022_1235_MOESM1_ESM.docx]

Additional File 1: Mplus Code for Power Analyses

**MSEM**

The following Mplus code can be used to estimate statistical power to detect mediation in a 3-2-1 model using MSEM. To run the analysis, users must provide input values of design parameters. These values are determined by the user based on their specific research application.

To input design parameters, users must replace the bracketed terms {} in the code below with either (a) a number representing the desired value of the design parameter, or (b) a number derived from the formula shown. All formulas are functions of the input design parameters (a3, b3, b2, c3, iccmM3, iccY2, iccY3, N1, N2, N3). Users must also indicate the number of replications to be performed for the Monte Carlo simulation. In this example, 500 reps are used.

***** MPLUS SCRIPT *****

**Varying parameters: a3, b3, b2, c3, iccmM3, iccY2, iccY3, N1, N2, N3**

TITLE: 3-2-1 mediation model, MLR;

MONTECARLO:

NAMES ARE x m y;

NOBS = {N3*N2*N1};

NCSIZES = 1[1];

CSIZES = {N3}[{N2}({N1})];

SEED = 347;

NREPS = 500;

BETWEEN = m (level3) x;

ANALYSIS: TYPE = THREELEVEL; ESTIMATOR = MLR;

MODEL POPULATION:

%BETWEEN level3%

m ON x*{a3*sqrt(iccm3)};

y ON m*{b3*sqrt(iccy3/iccm3)} x*{c3*sqrt(iccy3)};

x*1 m*{iccm3*(1-a3^2)} y*{iccy3*(1-b3^2-c3^2-2*a3*b3*c3)};

%BETWEEN level2%

y ON m*{b2*sqrt(iccy2/(1-iccm3))};

m*{1-iccm3} y*{iccy2*(1-b2^2)};

%WITHIN%

y*{1-iccy2-iccy3};

MODEL:

%BETWEEN level3%

m ON x*{a3*sqrt(iccm3)}(a3x);

y ON m*{b3*sqrt(iccy3/iccm3)} x*{c3*sqrt(iccy3)}(b3x c3x);

x*1 m*{iccm3*(1-a3^2)} y*{iccy3*(1-b3^2-c3^2-2*a3*b3*c3)}(vx3 vm3 vy3);

%BETWEEN level2%

y ON m*{b2*sqrt(iccy2/(1-iccm3))}(b2x);

m*{1-iccm3} y*{iccy2*(1-b2^2)}(vm2 vy2);

%WITHIN%

y*{1-iccy2-iccy3}(vy1);

MODEL CONSTRAINT: NEW(za3*{a3} zb3*{b3} zb2*{b2} indeff*{a3*b3}); !indeff = indirect effect

za3=a3x*sqrt(vx3)/sqrt(vm3+a3x^2*vx3);

zb3=b3x*sqrt(vm3+a3x^2*vx3)/sqrt(vy3+b3x^2*(vm3+a3x^2*vx3)+c3x^2*vx3+2*b3x*c3x*a3x*vx3);

zb2=b2x*sqrt(vm2)/sqrt(vy2+b2x^2*vm2);

indeff=za3*zb3;

*****end Mplus script*****

**Interpretation**

In the MODEL RESULTS section, under “New/Additional Parameters,” the estimate of power for the indirect effect will be listed in the last column of the first row as shown in the example output below. The parameter will be labeled “Indeff” (indirect effect). The power estimate for this example is highlighted by the box. In this example, empirically estimated power to detect mediation = 0.433.

MODEL RESULTS

ESTIMATES S. E. M. S. E. 95% % Sig

Population Average Std. Dev. Average Cover Coeff

New/Additional Parameters

Indeff 0.325 0.3679 0.2266 0.2165 0.0532 0.917 **0.433**

ICCM3 0.100 0.1002 0.0339 0.0296 0.0011 0.885 0.960

ICCY3 0.100 0.0999 0.0113 0.0109 0.0001 0.927 0.999

ICCY2 0.050 0.0499 0.0056 0.0053 0.0000 0.917 1.000

**MVM**

The following Mplus code can be used to estimate statistical power to detect mediation in a 3-2-1 model using standard multilevel linear regression based on manifest variables (MVM). This approach incorporates the centered within context with means reintroduced approach described by Zhang et al. (2009) in order to obtain correct (unconflated) estimates of the indirect effect. To run the simulation, users must provide values of the input design parameters. These values are determined by the user based on their specific research application. There are three main steps:

**Step 1: Generate N datasets for the simulation.**

Use Mplus to run stage 1 of an external Monte Carlo simulation. This will generate and save N datasets to be used for the power analysis. In the example below, 500 datasets are generated and saved; they are named rep0001-rep500.

**Step 2: Process the N datasets to include cluster means and within-cluster scores for the mediator.**

Using an external program, read the N datasets and in each find the column for M and the level-3 unit identifier. Compute level-3 cluster means M_k and add these to the data set. Also compute cluster-mean-centered scores (M_jk - M_k) and add these to data set. Save the modified datasets. In the example below, 500 datasests are modified and saved (rep0001-rep500).

**Step 3: Read N datasets into Mplus and calculate statistical power for mediation.**

Use Mplus to run stage 2 of an external Monte Carlo simulation. Read the N datasets into Mplus and run the specified analytic model. In our example below, 500 data sets (rep0001-rep500 files) are read into Mplus and the specified analytic model is estimate for each. Results of those analyses are automatically compiled and tabulated by Mplus.

In the “Detailed Steps” section below, please see examples Mplus code and additional information for each step.

**Detailed Steps:**

**Step 1: Generate N datasets for the simulation (example uses N=500)**

Replace all of the bracketed terms {} with either (a) a number representing the desired value of the parameter, or (b) a number derived from the formula shown. All formulas are functions of the input design parameters (a3, b3, b2, c3, iccmM3, iccY2, iccY3, N1, N2, N3). Indicate the number of replications to be performed for the Monte Carlo simulation. In this example, 500 reps are used.

***** MPLUS SCRIPT FOR STAGE 1 OF EXTERNAL MONTE CARLO *****

**Varying parameters: a3, b3, b2, c3, iccmM3, iccY2, iccY3, N1, N2, N3.**

TITLE: 3-2-1 mediation model, MLR;

MONTECARLO:

NAMES ARE x m y;

NOBS = {N3*N2*N1};

NCSIZES = 1[1];

CSIZES = {N3}[{N2}({N1})];

SEED = 347;

NREPS = 500;

BETWEEN = m (level3) x;

REPSAVE = ALL;

SAVE = rep*.dat;

ANALYSIS: TYPE = THREELEVEL; ESTIMATOR = MLR;

MITERATIONS = 1;

MODEL POPULATION:

%BETWEEN level3%

m ON x*{a3*sqrt(iccm3)};

y ON m*{b3*sqrt(iccy3/iccm3)} x*{c3*sqrt(iccy3)};

x*1 m*{iccm3*(1-a3^2)} y*{iccy3*(1-b3^2-c3^2-2*a3*b3*c3)};

%BETWEEN level2%

y ON m*{b2*sqrt(iccy2/(1-iccm3))};

m*{1-iccm3} y*{iccy2*(1-b2^2)};

%WITHIN%

y*{1-iccy2-iccy3};

MODEL:

%BETWEEN level3%

x m y; [x m y];

%BETWEEN level2%

m y;

%WITHIN%

y;

OUTPUT: NOCHISQUARE;

*****end Mplus script*****

**Step 2: Process N datasets by adding cluster means and within-cluster scores for the mediator (example uses N=500)**

EXAMPLE

Step 1 will produce N datasets that have columns x, m, y, id2, and id3. In our example, 500 datasets are generated. In this step, the user must process these datasets by adding the cluster means and cluster-mean-centered scores for m. To do this, first, add columns m3 and m2 to each of the N datasets. Second, calculate cluster means of m and enter them into column m3; these are calculated as the mean of m for each id3. Third, calculate the cluster-mean-centered scores for m and enter them into column m2; these are calculated as m-m3.

**Step 3: Read N datasets into Mplus and calculate statistical power for mediation.**

In this step, the 500 processed datasets are read back into Mplus and analyzed to produce the power estimate. Just as above, the varying parameters are a3, b3, b2, c3, iccmM3, iccY2, iccY3, N1, N2, N3. Replace all of the bracketed terms {} with either (a) a constant representing the desired value of the parameter, or (b) an integer derived from the formula shown. All formulas are functions of the input parameters (a3, b3, b2, c3, iccmM3, iccY2, iccY3, N1, N2, N3). Indicate the number of replications to be performed for the Monte Carlo simulation. In this example, 500 reps are used.

***** MPLUS SCRIPT FOR STAGE 2 OF EXTERNAL MONTE CARLO *****

**Varying parameters: a3, b3, b2, c3, iccmM3, iccY2, iccY3, N1, N2, N3.**

TITLE: 3-2-1 mediation model, MLR;
DATA: FILE = replist.dat; TYPE = MONTECARLO;
VARIABLE: NAMES = x m y id2 id3 m3 m2;
USEVARIABLES = x y m3 m2;
CLUSTER = id3 id2;
BETWEEN = m2 (id3) x m3;
ANALYSIS: TYPE = THREELEVEL; ESTIMATOR = MLR;

MODEL:

%BETWEEN id3%

m3 ON x*{a3*sqrt(iccm3)}(a3x);

y ON m3*{b3*sqrt(iccy3/iccm3)} x*{c3*sqrt(iccy3)} (b3x c3x);

x*1 m3*{iccm3*(1-a3^2)} y*{iccy3*(1-b3^2-c3^2-2*a3*b3*c3)}(vx3 vm3 vy3);

%BETWEEN id2%

y ON m2*{b2*sqrt(iccy2/(1-iccm3))}(b2x);

m2*{1-iccm3} y*{iccy2*(1-b2^2)}(vm2 vy2);

%WITHIN%

y*{1-iccy2-iccy3}(vy1);

MODEL CONSTRAINT: NEW(za3*{a3} zb3*{b3} zb2*{b2} indeff*{a3*b3}); !indeff = indirect effect

za3=a3x*sqrt(vx3)/sqrt(vm3+a3x^2*vx3);

zb3=b3x*sqrt(vm3+a3x^2*vx3)/sqrt(vy3+b3x^2*(vm3+a3x^2*vx3)+c3x^2*vx3+2*b3x*c3x*a3x*vx3);

zb2=b2x*sqrt(vm2)/sqrt(vy2+b2x^2*vm2);

indeff=za3*zb3;

*****end Mplus script*****

**Interpretation**

Results of the MVM power analysis are interpreted in the same way as the MSEM analysis. In the MODEL RESULTS section of the Mplus output, under “New/Additional Parameters,” the estimate of power for the indirect effect will be listed in the last column of the first row as shown in the example output example above. The parameter will be labeled “Indeff” (indirect effect). The Mplus output indicates the proportion of reps for which the parameter was statistically significant at *p* < .05, which represents the empirical power estimate.
